# Association of Posttraumatic Headache With Symptom Burden After Concussion in Children

**DOI:** 10.1001/jamanetworkopen.2023.1993

**Published:** 2023-03-08

**Authors:** Jacqueline Josee van Ierssel, Ken Tang, Miriam Beauchamp, Natalie Bresee, Achelle Cortel-LeBlanc, William Craig, Quynh Doan, Jocelyn Gravel, Todd Lyons, Rebekah Mannix, Serena Orr, Roger Zemek, Keith Owen Yeates

**Affiliations:** 1 Independent statistical consultant; 2 Department of Psychology, Université de Montréal, Montréal, Québec, Canada; 3 CHU Sainte-Justine Hospital Research Centre, Montreal, Québec, Canada; 4 Department of Pediatrics, Children’s Hospital of Eastern Ontario, Ottawa, Ontario, Canada; 5 University of Ottawa, Ottawa, Canada; 6 Department of Emergency Medicine, Children’s Hospital of Eastern Ontario, Ottawa, Ontario, Canada; 7 Faculty of Medicine, University of Ottawa, Queensway Carleton Hospital, Ottawa, Canada; 8 Department of Pediatrics, University of Alberta, Edmonton, Canada; 9 Stollery Children’s Hospital, Edmonton, Canada; 10 Department of Pediatrics, University of British Columbia, Vancouver, Canada; 11 BC Children’s Hospital Research Institute , Vancouver, Canada; 12 Department of Pediatric Emergency Medicine, Université de Montréal, Montréal, Canada; 13 Division of Emergency Medicine, Boston Children’s Hospital, Boston, Massachusetts; 14 Department of Pediatrics, Harvard Medical School, Boston, Massachusetts; 15 Department of Emergency Medicine, Harvard Medical School, Boston, Massachusetts; 16 Department of Pediatrics, University of Calgary, Calgary, Canada; 17 Alberta Children’s Hospital Research Institute, Calgary, Canada; 18 Department of Clinical Neurosciences, University of Calgary, Calgary, Canada; 19 Children’s Hospital of Eastern Ontario Research Institute, Ottawa, Canada; 20 Department of Psychology, University of Calgary, Calgary, Canada; 21 Hotchkiss Brain Institute, Calgary, Canada

## Abstract

**Importance:**

Headache is the most common symptom after pediatric concussion.

**Objectives:**

To examine whether posttraumatic headache phenotype is associated with symptom burden and quality of life 3 months after concussion.

**Design, Setting, and Participants:**

This was a secondary analysis of the Advancing Concussion Assessment in Pediatrics (A-CAP) prospective cohort study, conducted September 2016 to July 2019 at 5 Pediatric Emergency Research Canada (PERC) network emergency departments. Children aged 8.0-16.99 years presenting with acute (<48 hours) concussion or orthopedic injury (OI) were included. Data were analyzed from April to December 2022.

**Exposure:**

Posttraumatic headache was classified as migraine or nonmigraine headache, or no headache, using modified International Classification of Headache Disorders, 3rd edition, diagnostic criteria based on self-reported symptoms collected within 10 days of injury.

**Main Outcomes and Measures:**

Self-reported postconcussion symptoms and quality-of-life were measured at 3 months after concussion using the validated Health and Behavior Inventory (HBI) and Pediatric Quality of Life Inventory–Version 4.0 (PedsQL-4.0). An initial multiple imputation approach was used to minimize potential biases due to missing data. Multivariable linear regression evaluated the association between headache phenotype and outcomes compared with the Predicting and Preventing Postconcussive Problems in Pediatrics (5P) clinical risk score and other covariates and confounders. Reliable change analyses examined clinical significance of findings.

**Results:**

Of 967 enrolled children, 928 (median [IQR] age, 12.2 [10.5 to 14.3] years; 383 [41.3%] female) were included in analyses. HBI total score (adjusted) was significantly higher for children with migraine than children without headache (estimated mean difference [EMD], 3.36; 95% CI, 1.13 to 5.60) and children with OI (EMD, 3.10; 95% CI, 0.75 to 6.62), but not children with nonmigraine headache (EMD, 1.93; 95% CI, −0.33 to 4.19). Children with migraine were more likely to report reliable increases in total symptoms (odds ratio [OR], 2.13; 95% CI, 1.02 to 4.45) and somatic symptoms (OR, 2.70; 95% CI, 1.29 to 5.68) than those without headache. PedsQL-4.0 subscale scores were significantly lower for children with migraine than those without headache only for physical functioning (EMD, −4.67; 95% CI, −7.86 to −1.48).

**Conclusions and Relevance:**

In this cohort study of children with concussion or OI, those with posttraumatic migraine symptoms after concussion had higher symptom burden and lower quality of life 3 months after injury than those with nonmigraine headache. Children without posttraumatic headache reported the lowest symptom burden and highest quality of life, comparable with children with OI. Further research is warranted to determine effective treatment strategies that consider headache phenotype.

## Key Points

**Question:**

Is posttraumatic headache phenotype associated with symptom burden and quality of life 3 months after concussion among children aged 8 to 16 years?

**Findings:**

In this cohort study among 928 children with concussion or orthopedic injury, children with posttraumatic migraine symptoms had higher symptom burden and lower quality of life 3 months after concussion than children with nonmigraine headache. Children with no headache after concussion had the lowest symptom burden and highest quality of life following concussion, comparable with children with orthopedic injury.

**Meaning:**

These findings suggest that postacute headache phenotype was associated with symptom burden and quality of life 3 months after concussion.

## Teaser

This cohort study examines whether posttraumatic headache phenotype is associated with symptom burden and quality of life 3 months after concussion in children aged 8 to 16 years.

## Introduction

Approximately 840 000 children visit an emergency department (ED) in the US every year for a traumatic brain injury (TBI), 70% to 90% of which are considered a concussion.^1,2^ Although most children recover quickly, approximately one-third will continue to report symptoms beyond 1 month.^3,4^ Posttraumatic headache (PTH) occurs in up to 90% of children, most commonly with migraine features.^3,5,6,7^ Prior studies have identified several risk factors associated with prolonged recovery in children, including preinjury history of migraine, female sex, and increasing age, as well as past concussion with symptoms lasting more than 1 week.^3^ Emerging research suggests that PTH with migraine features is associated with persisting symptoms after concussion; however, previous studies are limited by small sample sizes, samples consisting only of athletes or recruited from specialty clinics beyond 1 month after injury, and lack of an orthopedic injury (OI) comparison group.^8,9,10^ Moreover, little is known about whether the postacute PTH phenotype is independently associated with prolonged recovery after controlling for known risk factors.

The primary objective of this study was to determine whether the postacute PTH phenotype is associated with total symptom burden, compared with the validated Predicting and Preventing Postconcussive Problems in Pediatrics (5P) clinical risk score,^3^ 3 months after acute concussion in children aged 8 to 16.99 years. We hypothesized that total symptom burden would be higher in children with a PTH migraine (PTH-M) phenotype compared with children with a PTH nonmigraine (PTH-NM) phenotype or without PTH after concussion, or compared with children with an OI without headaches. Secondary objectives were to determine whether PTH phenotype is associated with cognitive and somatic symptoms and total health-related quality-of-life and its components (physical, emotional, social, and school).

## Methods

### Design and Setting

This cohort study was approved by each participating site’s institutional review board. Parents or guardians and participants provided informed consent or assent as appropriate. This study is reported in accordance with the Strengthening the Reporting of Observational Studies in Epidemiology (STROBE) reporting guideline. This study was a secondary analysis of the Advancing Concussion Assessment in Pediatrics (A-CAP) prospective cohort study.^11^ Participants were recruited from 5 EDs within the Pediatric Emergency Research Canada (PERC) network (British Columbia Children’s Hospital, Vancouver; Alberta Children’s Hospital, Calgary; Stollery Children’s Hospital, Edmonton; Children’s Hospital of Eastern Ontario [CHEO], Ottawa; and Ste-Justine Hospital, Montreal) between 2016 and 2018.

### Participants

Participants were children aged 8.00 to 16.99 years who presented to a participating ED within 48 hours of injury. Children were eligible for inclusion in the concussion group if they had a history of blunt head trauma resulting in at least 1 of 3 criteria consistent with the WHO definition of mild TBI^12^: (1) an observed loss of consciousness less than 30 minutes; (2) a Glasgow Coma Scale score of 13 or 14; or (3) at least 1 acute sign or symptom of concussion as noted by ED medical personnel on a standard case report form. Inclusion criteria for the OI group were upper or lower extremity injuries (eg, fractures, sprains, or strains) arising from blunt trauma associated with Abbreviated Injury Scale (AIS)^13^ score of 4 or less. Exclusion criteria for the concussion group were deteriorating neurological status, neurosurgical intervention, loss of consciousness more than 30 minutes, posttraumatic amnesia more than 24 hours, or AIS score greater than 4. Exclusion criteria for the OI group were any head trauma or acute signs or symptoms of concussion (including headache) at the time of recruitment, surgical intervention, or procedural sedation. Exclusion criteria for both groups included previous overnight hospitalization for TBI, past concussion within 3 months, or a neurodevelopmental disorder.

### Protocol

Details of the A-CAP study have been published elsewhere.^11^ Briefly, using standardized questionnaires collected by trained research assistants via Research Electronic Data Capture (REDCap; Vanderbilt University)^14,15^ at ED enrollment, participants provided information on demographics, injury characteristics, previous concussion and migraine history, and the validated 5P risk score (ED-collected variables that stratify risk of postconcussion symptoms at 4-weeks).^3^ Parents identified their child’s race from predetermined categories (including Asian, Black, Hispanic, Indigenous, White, and other or multiracial) on an electronic questionnaire. Race and ethnicity were assessed since they have been reported as associated with prolonged recovery in adolescents after concussion.^16^ Parents rated their child’s preinjury symptoms retrospectively using the Health and Behavior Inventory (HBI).^17^ Children rated postinjury symptoms at a postacute visit targeted for 10 days after injury using the HBI^17^ and Postconcussion Symptom Interview,^18^ which were used to classify headache phenotype. Children completed the HBI^17^ and Pediatric Quality of Life Inventory–Version 4.0 (PedsQL-4.0)^19^ at a follow-up visit 3 months after injury.

### Independent Variable

#### Headache Phenotype Classification

Participants were considered to have a PTH if they endorsed a headache sometimes or often on the HBI^17^ within 10 days after injury. Headaches were classified into 1 of 4 mutually exclusive phenotype groups according to modified International Classification of Headache Disorders, 3rd Edition (ICHD-3)^20^ clinical criteria, based on self-reported symptoms: (1) PTH-M: participant with concussion endorsed PTH on the HBI^17^ and nausea or both photophobia and phonophobia on the Postconcussion Symptom Interview^18^; (2) PTH-NM: participant with concussion endorsed PTH that did not include migraine characteristics; (3) no PTH: participant with concussion endorsed PTH never or rarely, regardless of whether they endorsed nausea, photophobia, or phonophobia; (4) OI: participant with OI met the criteria for no PTH.

#### 5P Clinical Risk Score

The 5P clinical risk score was derived and validated in a large pediatric sample using variables measured in the ED to project the risk of postconcussion symptoms at 4 weeks.^3^ The 12-point score estimates risk based on age, sex, history of past concussion and maximum symptom duration, physician-diagnosed migraine history, answering questions slowly on clinical examination, tandem stance balance, and symptoms of headache, sensitivity to noise, and fatigue.

### Measures and Outcomes

#### Symptoms

The primary outcome was the total child-reported symptom score on the HBI^17^ at 3 months after injury. The HBI is a 20-item valid and reliable measure of postconcussion symptoms in children^17^ recommended by the National Institutes of Health (NIH)^21^ as a core common data element in the subacute (>3 days) and chronic (>3 months) postinjury intervals. Symptom frequency is rated on a 4-point scale (range, 0-3; 0 indicates never; 1, rarely; 2, sometimes; and 3, often) yielding a total score ranging from 0 to 60; higher scores indicate higher symptom burden. Secondary outcomes were separate subscale scores for cognitive (11 items) and somatic (9 items) symptoms.

#### Quality of Life

A secondary outcome was health-related quality of life, measured using the child-reported PedsQL-4.0,^22^ which is associated with postconcussion symptoms at 3 months.^23^ The PedsQL-4.0^22^ has demonstrated excellent reliability and validity^19,24^ and is recommended as a supplemental highly recommended common data element by the NIH.^21^ It is a 23-item questionnaire using a 5-point rating scale (range, 0-4; 0 indicates never a problem; 4, almost always a problem) that yields a total score and separate subcomponent scores for physical activity (8 items), emotional functioning (5 items), social functioning (5 items), and school functioning (5 items).^22^ Item scores are transformed into a scale of 0 to 100; higher scores represent higher quality of life.

#### Reliable Change

We assessed reliable change by regressing postinjury symptom scores for child ratings onto parents’ retrospective preinjury symptom scores separately for the HBI total and subscale scores. The resulting regression coefficients were used to compute standardized change scores by subtracting estimated scores from actual postinjury scores and dividing by the SE of the estimate. Standardized change scores exceeding 1.64 (ie, in the extreme 5% of the distribution) were considered to indicate a reliable increase in symptoms.^25^

### Statistical Analysis

Initial postinjury participant characteristics were summarized according to headache phenotype using descriptive statistics (eg, medians, frequencies). Differences in initial postinjury participant characteristics among headache phenotype groups were assessed using Kruskal-Wallis tests for continuous variables or χ^2^ tests for categorical variables.

Multivariable statistical models were fitted to assess the association between headache phenotype and each of the 11 study outcomes, adjusting for known confounders of prolonged recovery: age, sex, migraine history, past concussion maximum symptom duration, and total 5P risk score,^4,18^ as well as additional covariates of interest: race, parental educational attainment, social deprivation index, material deprivation index, family history of migraine, and preinjury HBI cognitive and somatic scores.^16,26^ All covariates were retained in the final model. For each of the 8 continuous outcomes (HBI^17^ and PedsQL^22^ total and subscale scores), multivariable linear regressions were fitted. For each of the 3 binary outcomes (HBI total and subscale reliable change scores), multivariable logistic regressions were fitted. As post hoc exploratory analyses, we refitted all PedsQL models using a modified 5P risk score that removed symptoms common to our headache phenotype classification (headache, phonophobia) instead of the original total 5P risk score.

To minimize potential biases due to missing data, our modeling procedure included an initial multiple imputation step that applied a chained equation approach with additive regression, bootstrapping, and estimated mean matching techniques (eMethods in Supplement 1).^27^ Bonf-Holm corrections were applied to the *P* values, and 2-tailed significance was set at *P* < .05. All analyses were performed using R statistical software version 4.2.2 (R Project for Statistical Computing). Data were analyzed from April to December 2022.

## Results

Of 3051 children meeting eligibility criteria, 967 provided consent or assent and were enrolled, and 928 (median [IQR] age, 12.2 [10.5-14.3] years; 383 [41.3%] female) were included in this study. Of those 928 participants, 548 children with concussion, including 254 (46.4%) with PTH-M, 134 (24.5%) with PTH-NM, and 160 (29.2%) with no PTH, and 239 OI without headache (45% female) returned for the postacute visit. Postacute visits were conducted a median (IQR) of 8 (6-10) days later, at which point participants were classified into headache phenotypes (eFigure in Supplement 1). A total of 180 participants were unclassifiable due to incomplete survey data or lack of postacute assessment. Several initial postinjury participant characteristics differed significantly among headache phenotype groups (Table 1). Compared with children with PTH-NM, no PTH, or OI, participants with PTH-M were more often female and White, were more likely to report past concussion symptoms longer than 1 week, had a higher 5P risk score at the initial ED visit, reported higher preinjury symptoms, and had lower scores on some aspects of preinjury quality of life (Table 1). Children with no PTH were younger than all other groups. Initial postinjury participant characteristics were similar between those who were and were not classifiable for headache phenotype, except for somatic symptoms (eTable 1 in Supplement 1).

**Table 1.** Participant Initial Postinjury Characteristics

Variable	OI (no headache) (n = 239)	Concussion			Overall (N = 787)	Group difference, *P* value
		No headache (n = 160)	Nonmigraine headache (n = 134)	Migraine headache (n = 254)		
Site						
Calgary	52 (21.8)	41 (25.6)	36 (26.9)	53 (20.9)	182 (23.1)	.01
Edmonton	48 (20.0)	26 (16.3)	23 (17.2)	60 (23.6)	157 (19.9)	
Montreal	19 (7.9)	18 (11.3)	18 (13.4)	22 (8.9)	77 (9.8)	
Ottawa	43 (18.0)	34 (21.3)	31 (23.1)	72 (28.3)	180 (22.9)	
Vancouver	77 (32.2)	41 (25.6)	26 (19.4)	47 (18.5)	191 (24.3)	
Time from injury to ED visit, median (IQR), h (n = 787)	6 (3-22)	4 (2-15)	6 (3-10)	4 (3-18_	5 (3-20)	.004
Time from injury to postacute visit, median (IQR), d (n = 786)	9 (7-10)	8 (6-10)	8 (6-10)	8 (6-10)	8 (6-10)	.02
Time from injury to 3-mo assessment, median (IQR), d (n = 697)	94 (89-102)	94 (90-101)	94 (88-100)	97 (91-104)	95 (89-102)	.11
Age, median (IQR), y (n = 787)	13 (11-14)	11 (10-13)	13 (10-15)	13 (11-15)	12 (11-14)	<.001
Sex (n = 787)						
Male	132 (55.2)	114 (71.3)	90 (67.2)	126 (49.6)	462 (58.7)	<.001
Female	107 (44.8)	46 (28.8)	44 (32.8)	128 (50.4)	325 (41.3)	
Race (n = 781)						
Asian	20 (8.4)	26 (16.3)	16 (11.9)	9 (3.5)	71 (9.0)	.008
Black	7 (2.9)	5 (3.1)	6 (4.5)	10 (3.9)	28 (3.6)	
Hispanic	10 (4.2)	5 (3.1)	2 (1.5)	5 (2.0)	22 (2.8)	
Indigenous	2 (0.8)	6 (3.8)	2 (1.5)	5 (2.0)	15 (1.9)	
White	166 (69.5)	100 (62.5)	93 (69.4)	186 (73.2)	545 (69.3)	
Other or multiracial	34 (14.2)	16 (10.0)	14 (10.4)	36 (14.2)	100 (12.7)	
Parental education (n = 772)						
≤High school	34 (14.2)	29 (18.1)	19 (14.2)	36 (14.2)	118 (15.0)	.53
Trade or some college	69 (28.9)	40 (25.0)	40 (29.5)	80 (31.5)	229 (29.1)	
Bachelor’s degree	83 (34.7)	67 (41.9)	49 (36.6)	84 (33.1)	283 (36.0)	
>Bachelor’s degree	49 (20.5)	21 (13.1)	24 (17.9)	48 (18.9)	142 (18.0)	
Social deprivation index, median (IQR), percentile (n = 760)	43 (24-67)	40 (24-64)	40 (25-66)	38 (21-63)	40 (23-65)	.52
Material deprivation index, median (IQR), percentile (n = 760)	23 (12-53)	30 (13-56)	25 (10-53)	26 (11-54)	26 (11-54)	.71
Past concussion maximum symptom duration (n = 780)						
<1 wk or no previous concussions	201 (84.1)	139 (86.9)	109 (81.3)	188 (74.0)	637 (80.9)	.007
≥1 wk	36 (15.1)	20 (12.5)	25 (18.9)	62 (24.4)	143 (18.2)	
History of migraine headaches (n = 777)	9 (3.8)	7 (4.4)	8 (6.0)	18 (7.1)	42 (5.3)	.34
Family history of migraine headaches (n = 785)	83 (34.7)	53 (33.4)	46 (34.3)	109 (42.9)	291 (37.0)	.12
5P risk score at ED visit, median (IQR) (n = 781)	4 (2-5)	5 (4-7)	6 (5-7)	7 (6-8)	5 (4-7)	<.001
Preinjury HBI score, median (IQR) (n = 785)^a^						
Total	7 (1-15)	10 (3-19)	8 (3-18)	10 (4-19)	9 (3-17)	<.001
Somatic	1 (0-3)	1 (0-2)	1 (0-4)	2 (1-5)	1 (0-4)	<.001
Cognitive	5 (0-11)	10 (2-16)	7 (1-14)	8 (2-14)	7 (2-14)	.002
Preinjury PedsQL-4.0 score, median (IQR) (n = 785)^a^						
Total	87 (7-93)	85 (74-94)	85 (76-91)	84 (73-92)	85 (75-93)	.12
Physical activity	94 (84-100)	97 (88-100)	94 (85-100)	94 (81–100)	94 (84-100)	.10
Emotional functioning	85 (70-95)	80 (65-95)	80 (65-90)	75 (60-90)	80 (65-95)	.003
Social functioning	95 (78-100)	90 (75-100)	90 (75-100)	90 (75-100)	90 (75-100)	.14
School functioning	80 (65-95)	75 (65-90)	80 (60-90)	75 (60-90)	80 (65-90)	.04

Abbreviations: 5P, Predicting and Preventing Postconcussive Problems in Pediatrics; ED, emergency department; HBI, Health and Behavior Inventory; OI, orthopedic injury; PedsQL-4.0, Pediatric Quality of Life–Version 4.0.

^a^ Parent-rated.

### Symptom Burden

**Figure 1.**
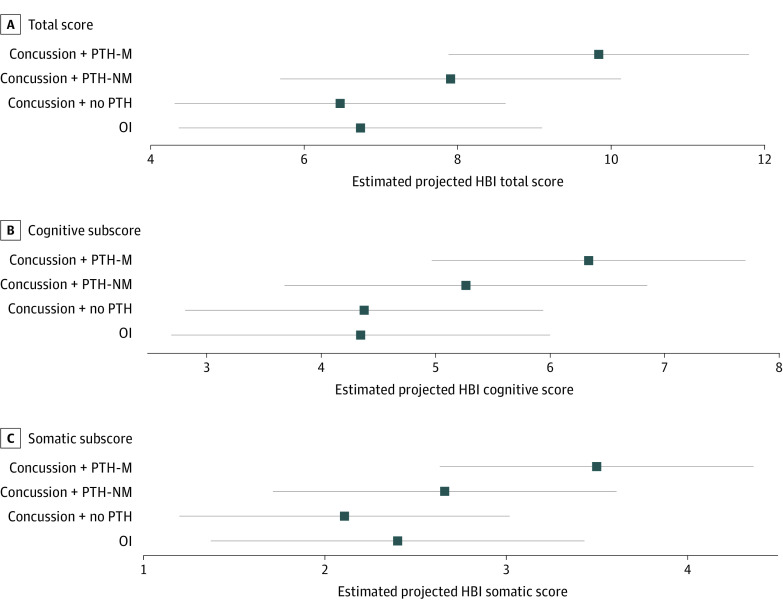
Estimated Child-Reported Health and Behavior Inventory (HBI) Scores at 3 Months Points represent estimated means from multivariable model adjusting for age, sex, race, parental education, social deprivation index, material deprivation index, past concussion maximum symptom duration, migraine history, family migraine history, Predicting and Preventing Postconcussive Problems in Pediatrics risk score, and preinjury HBI cognitive and somatic scores. Whiskers represent the 95% CI of the estimated means. OI indicates orthopedic injury; PTH-M, posttraumatic headache migraine phenotype; PTH-NM, posttraumatic headache nonmigraine phenotype.

Posttraumatic migraine phenotype was significantly associated with total symptom burden in a multivariable model even after including 5P risk score (estimated mean difference [EMD], 3.10; 95% CI, 0.75 to 5.44; *P* = .01) (Table 2). The 5P risk score was significant at a univariate level but not in the multivariable model (EMD, 0.26; 95% CI, −0.26 to 0.77; *P* = .32). The HBI total score (adjusted) was significantly higher for children with PTH-M than for those with no PTH (EMD, 3.36; 95% CI, 1.13-5.60) and for those with OI (EMD, 3.10; 95% CI, 0.75 to 5.44) (eTable 2 in Supplement 1). Mean HBI total scores were lower for children with PTH-NM compared with those with PTH-M (EMD, −1.93; 95% CI, 0.33 to −4.19), and higher compared with no PTH (EMD, 1.44; 95% CI, −0.95 to 3.92) and those with OI (EMD, 1.17; 95% CI, −1.29 to 3.63) (Figure 1A).

**Table 2.** Association of Posttraumatic Headache Phenotype With Total Symptom Burden (HBI Total Score) at 3 Months in a Multivariable Linear Regression^a^

Parameter estimates	HBI total		
	Coefficient (95% CI)	*t*	*P* value
Intercept	2.42 (−2.47 to 7.32)	0.97	.33
Headache phenotype			
Orthopedic injury	0 [Reference]	NA	NA
Concussion + no PTH	−0.27 (−2.60 to 2.07)	−0.22	.82
Concussion + PTH-NM	1.17 (−1.29 to 3.63)	0.93	.35
Concussion + PTH-M	3.10 (0.75 to 5.44)	2.59	.01
Age	0.02 (−0.29 to 0.33)	0.14	.89
Sex			
Male	0 [Reference]		
Female	2.54 (0.81 to 4.28)	2.87	.004
Race			
Asian	3.81 (1.17 to 6.44)	2.84	.005
Black	4.88 (0.80 to 8.95)	2.35	.02
Hispanic	3.81 (−0.54 to 8.17)	1.72	.09
Indigenous	2.30 (−1.74 to 6.34)	1.12	.26
White	0 [Reference]		
Other or multiracial	1.05 (−1.26 to 3.35)	0.89	.37
Parental education			
≤High school	0 [Reference]		
Trade or some college	1.18 (−1.11 to 3.47)	1.01	.31
Bachelor’s degree	0.01 (−2.33 to 2.35)	0.01	.99
>Bachelor’s degree	−0.40 (−2.99 to 2.18)	−0.31	.76
Deprivation index, percentile			
Social	0.01 (−0.02 to 0.03)	0.52	.60
Material	0.01 (−0.2 to 0.04)	0.85	.40
Past concussion maximum symptom duration			
<1 wk or no previous concussions	0 [Reference]		
≥1 wk	0.04 (−1.95 to 2.02)	0.04	.97
Migraine history	1.44 (−1.99 to 4.86)	0.82	.41
Family migraine history	1.59 (0.07 to 3.11)	2.05	.04
5P risk score at ED visit	0.26 (−0.26 to 0.77)	0.99	.32
Preinjury HBI			
Cognitive subscore^b^	0.26 (0.16 to 0.36)	4.98	<.001
Somatic subscore^b^	0.34 (0.14 to 0.54)	3.41	<.001

Abbreviations: 5P, Predicting and Preventing Postconcussive Problems in Pediatrics; ED, emergency department; HBI, Health and Behavior Inventory; NA, not applicable; PTH, posttraumatic headache.

^a^ Model logistic regression: χ^2^_21_ = 146.62; *P* < .001; *R*^2^ = 0.15; adjusted *R*^2^ = 0.13; observed = 928.

^b^ Parent-reported.

At 3 months, children with PTH-M did not differ significantly on cognitive symptoms compared with those with PTH-NM (EMD, 1.07; 95% CI, −0.55 to 2.69), those with no PTH (EMD, 1.96; 95% CI, 0.37 to 3.55), or those with OI (EMD, 1.99; 95% CI, 0.36 to 3.62) (Figure 1B). Children with PTH-M reported significantly higher somatic symptoms than those with no PTH (EMD, 1.39; 95% CI, 0.43 to 2.36) (Figure 1C). Children with PTH-M did not differ significantly on somatic symptoms from those with PTH-NM (EMD, 0.84; 95% CI, −0.11 to 1.79) or those with OI (EMD, 1.10; 95% CI, 0.06 to 2.14). In addition to headache phenotype (*F* = 5.2), preinjury cognitive (*F* = 24.8) and somatic (*F* = 11.6) symptoms and sex (*F* = 8.3) were associated with total symptoms at 3 months.

### Quality of Life

**Figure 2.**
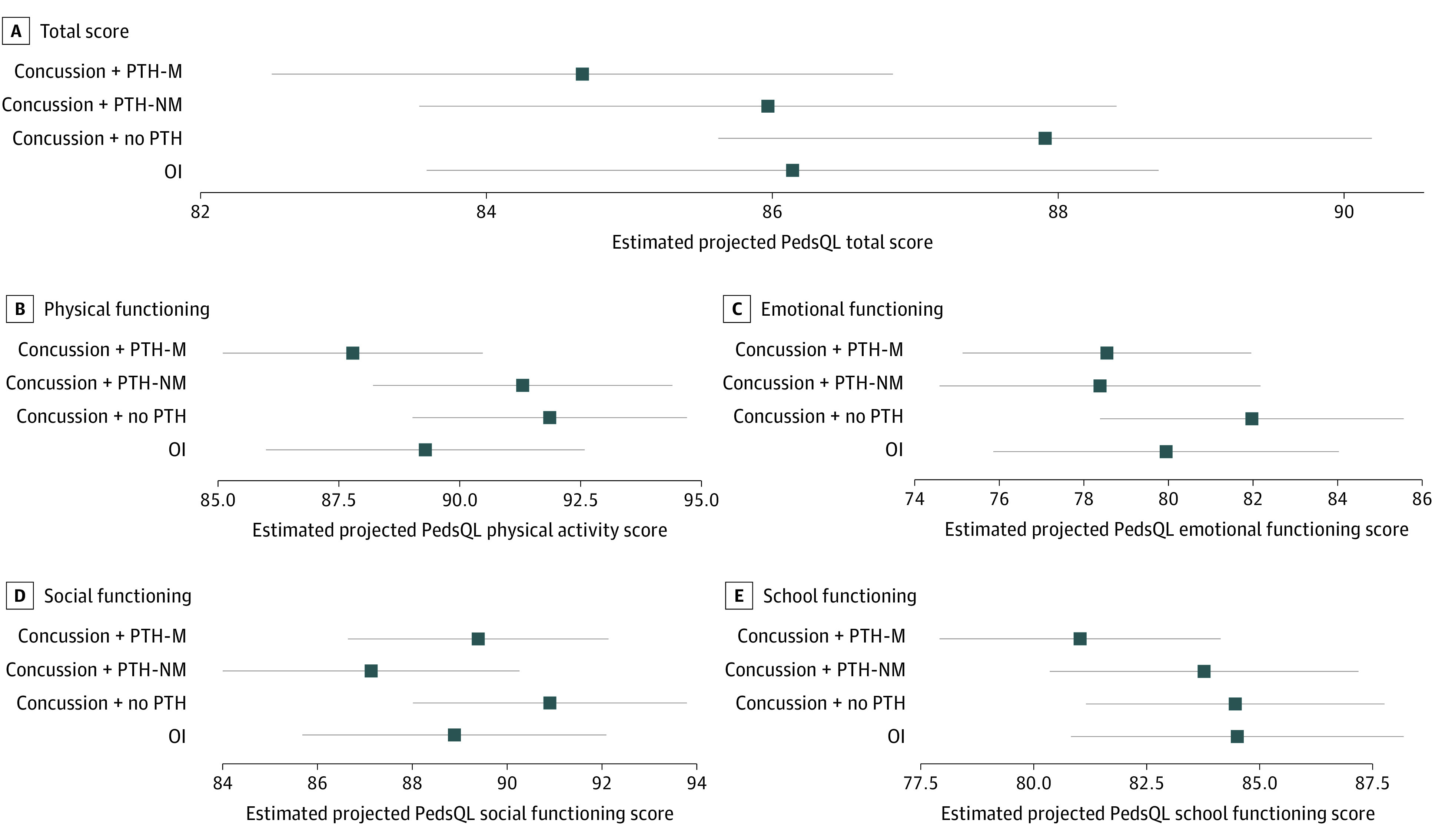
Estimated Child-Reported Pediatric Quality of Life–Version 4.0 (PedsQL-4.0) Scores at 3 Months Points represent estimated means from multivariable model adjusting for age, sex, race, parental education, social deprivation index, material deprivation index, past concussion maximum symptom duration, migraine history, family migraine history, Predicting and Preventing Postconcussive Problems in Pediatrics risk score, and preinjury Health and Behavior Inventory cognitive and somatic scores. Whiskers represent the 95% CI of the estimated means. OI indicates orthopedic injury; PTH-M, posttraumatic headache migraine phenotype; PTH-NM, posttraumatic headache nonmigraine phenotype.

Posttraumatic migraine phenotype was not significantly associated with total quality of life in a multivariable model (coefficient, −1.47; 95% CI, −4.07 to 1.12; *P* = .27) (eTable 3 in Supplement 1). The PTH-M group had a lower estimated mean (adjusted) scores on the PedsQL-4.0 physical activity subscale compared with the no PTH group (EMD, −4.09; 95% CI, −7.00 to −1.18; *P* = .04) (Figure 2; eTable 4 in Supplement 1). The PTH-M and PTH-NM groups did not differ significantly on total or PedsQL-4.0 subscale scores in multivariable models. Exploratory analyses using a modified 5P risk score found significant differences in PedsQL-4.0 scores between the PTH-M and no PTH groups for total (EMD, −3.49; 95% CI, −5.88 to −1.10; *P* = .03) and physical functioning (EMD, −4.32; 95% CI, −7.19 to −1.44; *P* = .02) scores, and between PTH-M and OI groups for school functioning (EMD, −4.55; 95% CI, −7.66 to −1.43; *P* = .03). Preinjury cognitive symptoms were strongly associated with total quality of life (*F* = 32.5) and emotional (*F* = 20.5), social (*F* = 25.0), and school (*F* = 38.4) functioning. Preinjury somatic symptoms and 5P risk score also were significantly associated with PedsQL-4.0 total and physical subscale scores.

### Reliable Change

Children with PTH-M were 2 times more likely to report reliable increases in total symptoms (odds ratio [OR], 2.13; 95% CI, 1.02 to 4.45) compared with the no PTH group. Furthermore, children with PTH-M were nearly 3 times more likely to report reliable increases in somatic symptoms (OR, 2.70; 95% CI, 1.29-5.68) compared with the no PTH group (Table 3).

**Table 3.** Reliable Change on the Child-Reported HBI at 3 Months Between Headache Phenotype Groups^a^

Headache phenotype	Odds ratio (95%CI)	Adjusted *P* value^a^
**HBI total score** ^b^		
Concussion + PTH-M vs concussion + PTH-NM	1.29 (0.67-2.51)	.90
Concussion + PTH-M vs concussion + no PTH	2.13 (1.02-4.45)	.26
Concussion + PTH-M vs OI	2.40 (0.96-6.01)	.31
Concussion + PTH-NM vs concussion + no PTH	1.65 (0.72-3.79)	.80
Concussion + PTH-NM vs OI	1.86 (0.72-4.78)	.80
Concussion + no PTH vs OI	1.13 (0.43-2.93)	.90
**HBI cognitive subscore** ^b^		
Concussion + PTH-M vs concussion + PTH-NM	1.02 (0.57-1.80)	>.99
Concussion + PTH-M vs concussion + no PTH	1.57 (0.84-2.94)	.96
Concussion + PTH-M vs OI	1.08 (0.58-1.99))	>.99
Concussion + PTH-NM vs concussion + no PTH	1.54 (0.78-3.07)	>.99
Concussion + PTH-NM vs OI	1.06 (0.54-2.08)	>.99
Concussion + no PTH vs OI	0.69 (0.36-1.32)	>.99
**HBI somatic subscore** ^b^		
Concussion + PTH-M vs concussion + PTH-NM	1.41 (0.72-2.75)	.65
Concussion + PTH-M vs concussion + no PTH	2.70 (1.29-5.68)	.05
Concussion + PTH-M vs OI	2.57 (1.04-6.34)	.21
Concussion + PTH-NM vs concussion + no PTH	1.92 (0.85-4.32)	.46
Concussion + PTH-NM vs OI	1.83 (0.70-4.73)	.65
Concussion + no PTH vs OI	0.95 (0.34-2.64)	.92

Abbreviations: HBI, Health and Behavior Inventory; OI, orthopedic injury; PTH-M, posttraumatic headache migraine phenotype; PTH-NM, posttraumatic headache nonmigraine phenotype.

^a^ Bonf-Holm–adjusted.

^b^ HBI total range: 0-60; cognitive range: 0-33; and somatic range: 0-27. Higher score indicates higher symptom burden.

## Discussion

This cohort study found that children with PTH migraine symptoms at 10 days after concussion had higher symptom burden and lower quality of life 3 months following concussion compared with children with PTH-NM symptoms, those with no headache, and children with OI. Children with no headache within 10 days after concussion had the best 3-month outcomes, comparable with children with OI. In keeping with our hypothesis, migraine phenotype remained associated with total symptoms even after adjusting for established risk factors for the development of persisting postconcussion symptoms^3^ but not consistently for quality of life.

Our findings extend preliminary evidence that headache phenotype is associated with symptom burden after pediatric concussion. In a prospective cohort study with more than 3000 children, Zemek et al^3^ found that preinjury migraine history, headache, and phonophobia identified in the ED were associated with persistent postconcussion symptoms at 4 weeks after injury in a multivariable model. Similarly, Kontos et al^8^ found that high school football players who experienced concussion and acute migraine headache were 7 times more likely to take longer than 20 days to recover compared with players with no headache and nearly 3 times more likely compared with players with nonmigraine headaches. Kamins et al^9^ found that posttraumatic migraine was associated with prolonged time to recovery compared with no migraine and no headache in children recruited from specialty concussion clinics within 8 weeks of injury. Similarly, Klein et al^10^ identified PTH migraine phenotype using the 3-item ID Migraine Screener in children presenting to a hospital brain injury program up to 1 year after concussion. Children with migraine phenotype took longer to recover than those with nonmigraine phenotype; the study did not include children with orthopedic injury or no PTH.

Importantly, the 5P risk score estimates the risk of persistent symptoms at 1 month based on demographic and clinical variables assessed within the first 48 hours after concussion. Our findings suggest that the type of headache matters in children who continue to report PTH at approximately 1 week after concussion. Because symptom improvement occurs primarily in the first week after injury,^28^ the children in our study who continued to report PTH at the 10-day postacute assessment represent a different group than those who recover quickly (ie, those with no PTH). Our findings may be generalizable to a broader population, given the large ED-recruited cohort with standardized postacute follow-up and long-term outcome assessment. Children who continue to experience symptoms 3 months after concussion often report a lower quality of life^23^; thus, our finding that children with migraine symptoms after concussion had a reliable increase in somatic symptoms is clinically important. While previous research has indicated that PTH-M was associated with objective neurocognitive performance within 2 weeks after concussion,^8^ we found cognitive symptoms at 3 months did not differ in children based on PTH phenotype.

In our analysis, preinjury symptoms and the 5P risk score were more consistently associated with quality of life than headache phenotype was. However, when we removed shared symptoms from the 5P risk score that were used to classify headache phenotype (headache and phonophobia) and used the modified score in a post hoc analysis, children PTH-M had significantly lower total quality of life and physical functioning than those with no PTH and lower school functioning than children with OI. Thus, quality of life may be more dependent on a child’s quantity of symptoms, rather than the specific symptoms themselves, since both a high 5P risk score and migraine classification require multiple symptoms. Alternatively, the shared migraine symptoms may be responsible for much of the overlap between the 5P and PTH groups, and this may explain why headache phenotype was not associated with quality of life, except for the physical subscale, in our multivariable models.

Classified as a secondary headache (ICHD-3),^20^ PTH shares similarities with primary headache disorders, although without identifying or defining symptoms, which can make clinical management challenging. The pathophysiological mechanism underlying PTH is unclear and likely multifactorial.^29,30,31^ Preliminary neuroimaging studies demonstrate significant differences between PTH and primary migraine in brain structure^32^ and in static and dynamic functional connectivity in regions responsible or pain processing.^33^ Our finding of significant differences in preinjury characteristics between headache phenotypes supports the adage that prognosis following concussion depends not just on the injury itself, but who gets injured.^34^ This may also explain why controlling for the 5P risk score, which accounts for age, sex, past concussion symptom maximum duration, and migraine history, attenuated some of the significant associations of headache phenotype with quality of life.

Multidisciplinary management of postconcussion symptoms often includes pharmacological intervention,^35,36^ physical and vestibular rehabilitation,^37,38,39^ subsymptom threshold aerobic exercise,^40,41^ and cognitive behavioral therapy,^42,43^ with varying effectiveness. However, limited evidence is available regarding the efficacy of pediatric PTH management.^44,45^ Our results suggest clinical management should target early intervention for children with migraine symptoms to reduce the risk of prolonged symptom burden. Further research to determine effective treatment strategies that consider headache phenotype is warranted.^44,45^

This study has several strengths. We enrolled a large prospective cohort and included an OI comparison group to assess the specific association of PTH phenotype with symptoms and quality of life after concussion. Confidence in our data is bolstered by the use of standardized data collection procedures by trained research assistants and rigorous validated outcome measures with good reliability. Our findings are likely generalizable to children with varying mechanisms of injury, since recruitment was not limited to sport-related concussion. By recruiting from pediatric EDs within 48 hours of injury, we reduced the confounding effect of time to initial assessment, since late clinical care is associated with prolonged recovery.^46^

### Limitations

The study has some limitations. First, we retrospectively classified PTH phenotype according to self-reported migraine characteristics using modified ICHD-3 criteria, rather than physician diagnosis. However, we included clinical characteristics that are consistent with ICHD-3^20^ diagnostic criteria and with previous studies.^8,9^ Furthermore, the percentage of children in our study with migraine was similar to previously published rates following pediatric concussions,^6,7^ suggesting we appropriately classified participants according to PTH phenotype. Second, unmeasured confounders, such as objective measures of vestibular dysfunction associated with prolonged recovery,^47^ may have influenced our estimates. Our large sample size permitted covarying for multiple known risk factors associated with prolonged recovery, including the 5P risk score, which mitigates this risk. Third, we did not measure or attempt to control for clinical interventions that could modify outcomes.

## Conclusions

In this cohort study of children with concussion or OI, those with PTH-M exhibited higher symptom burden and lower physical functioning quality of life 3 months after injury than those with PTH-NM. Children without PTH after concussion reported the lowest symptom burden and highest quality of life, similar to children with OI. Early identification of migraine after pediatric concussion may inform prognostication and targeted intervention to prevent prolonged recovery. Further research to determine effective treatment strategies that consider headache phenotype is warranted.

## Notes

Supplement 1.
eMethods.
**eFigure.** Flowchart of Participants Classified Into Posttraumatic Headache Phenotype Following Acute Concussion or Orthopedic Injury **eTable 1.** Comparison of Initial Postinjury Participant Characteristics Between Those Classifiable vs Not Classifiable Into Posttraumatic Headache Phenotype **eTable 2.** Total and Subscale Mean Differences on the Child-Reported HBI at 3 Months Among Headache Phenotype Groups **eTable 3.** Association of Posttraumatic Headache Phenotype With Quality of Life (PedsQL-4.0 Total Score) at 3 Months in a Multivariable Linear Regression **eTable 4.** Total and Subscale Mean Differences on the Child-Reported PedsQL-4.0 at 3 Months Among Headache Phenotype Groups

Supplement 2.
Pediatric Emergency Research Canada A-CAP Study Team Members

Supplement 3.
Data Sharing Statement
