# Supplementary material for: Association of Posttraumatic Headache With Symptom Burden After Concussion in Children
Source: JAMA Netw Open. 2023 Mar 8;6(3):e231993. doi: 10.1001/jamanetworkopen.2023.1993 (PMC9996395; doi:10.1001/jamanetworkopen.2023.1993)
Supplement: Supplement 1. — eMethods. eFigure. Flowchart of Participants Classified Into Posttraumatic Headache Phenotype Following Acute Concussion or Orthopedic Injury eTable 1. Comparison of Initial Postinjury Participant Characteristics Between Those Classifiable vs Not Classifiable Into Posttraumatic Headache Phenotype eTable 2. Total and Subscale Mean Differences on the Child-Reported HBI at 3 Months Among Headache Phenotype Groups eTable 3. Association of Posttraumatic Headache Phenotype With Quality of Life (PedsQL-4.0 Total Score) at 3 Months in a Multivariable Linear Regression eTable 4. Total and Subscale Mean Differences on the Child-Reported PedsQL-4.0 at 3 Months Among Headache Phenotype Groups [file jamanetwopen-e231993-s001.pdf]

Supplemental Online Content

van Ierssel JJ, Tang K, Beauchamp M, et al; Pediatric Emergency Research Canada A-CAP Study Team. Association of posttraumatic headache with symptom burden after concussion in children. *JAMA Netw Open*. 2023;6(3):e231993. doi:10.1001/jamanetworkopen.2023.1993

eMethods.
eFigure. Flowchart of Participants Classified Into Posttraumatic Headache Phenotype Following Acute Concussion or Orthopedic Injury
eTable 1. Comparison of Initial Postinjury Participant Characteristics Between Those Classifiable vs Not Classifiable Into Posttraumatic Headache Phenotype
eTable 2. Total and Subscale Mean Differences on the Child-Reported HBI at 3 Months Among Headache Phenotype Groups
eTable 3. Association of Posttraumatic Headache Phenotype With Quality of Life (PedsQL-4.0 Total score) at 3 Months in a Multivariable Linear Regression
eTable 4. Total and Subscale Mean Differences on the Child-Reported PedsQL-4.0 at 3 Months Among Headache Phenotype Groups

This supplemental material has been provided by the authors to give readers additional information about their work.

© 2023 van Ierssel JJ et al. *JAMA Network Open.*

eMethods. Multiple Imputation Modeling

To enhance the quality of data imputation, we broadly included 84 variables (all study outcomes, all covariates, plus auxiliary variables collected over the multiple timepoints of the A-CAP study) and all eligible observations (n=967) in the imputation process; 64 imputed datasets were generated to match the proportion of observations with any incomplete values on imputation variables.[1] To prepare these imputed datasets for statistical modeling, all OI patients with a definite headache (n=39) were excluded, leaving a final sample of 928 usable observations. Once final models were derived for each outcome, variance inflation factors for each parameter were assessed to verify the lack of significant collinearity (i.e., <5 expected). Then, post-model fit contrasts were performed to provide all possible pairwise comparisons of headache phenotype groups by estimating the (adjusted) mean differences for each outcome.

Reference

1. White I, Royston P, Wood A. Multiple imputation using chained equations: Issues and guidance for practice. *Stat Med*. 2011;30(4):377-399.

© 2023 van Ierssel JJ et al. *JAMA Network Open.*

eFigure. Flowchart of Participants Classified Into Posttraumatic Headache Phenotype Following Acute Concussion or Orthopedic Injury

OI, orthopaedic injury; *PTH*, posttraumatic headache

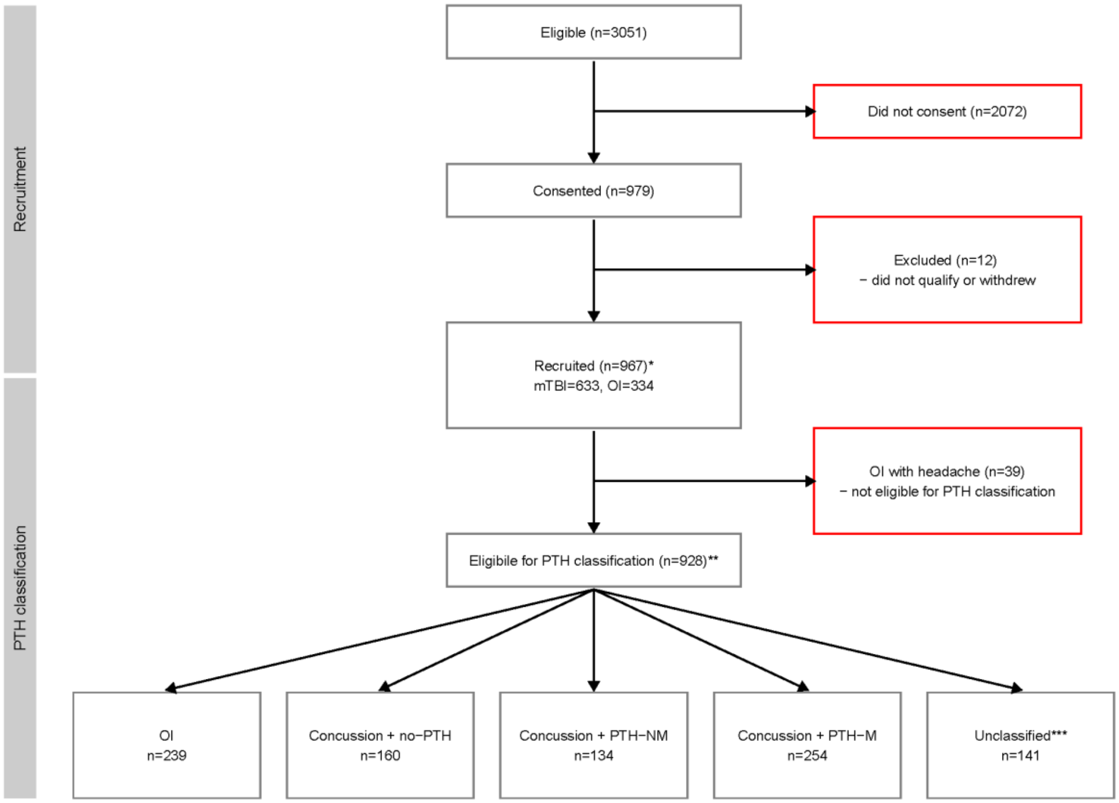

* *multiple imputation of missing data was performed at this stage to facilitate statistical modeling*
** *all observations contributed to the final statistical models*
*** *due to missing data from either incompleted survey items or non–participation of PA assessment*

© 2023 van Ierssel JJ et al. *JAMA Network Open.*

eTable 1. Comparison of Initial Post-Injury Participant Characteristics Between Those Classifiable vs Not Classifiable Into Posttraumatic Headache Phenotype[a]

Variable	Classifiable N=787 No. (%)	Not Classifiable N=180 No. (%)	p-value
**Site (Classifiable: n=787; Not Classifiable: n=180)**			0.39
Calgary	182 (23.1)	44 (24.4)	
Edmonton	157 (19.9)	27 (15.0)	
Montreal	77 (9.8)	23 (12.8)	
Ottawa	180 (22.9)	37 (20.6)	
Vancouver	191 (24.3)	49 (27.2)	
**Age, median (IQR), y (n=787; Not Classifiable: n=180)**	12.3 (10.6, 14.5)	12.3 (10.3, 14.0)	0.18
**Sex (n=787; Not Classifiable: n=180)**			0.44
Male	462 (58.7)	100 (55.6)	
Female	325 (41.3)	80 (44.4)	
**Race (n=781; Not Classifiable: n=53)**			0.64
Asian	71 (9.1)	4 (7.5)	
Black	28 (3.6)	0 (0.0)	
Indigenous	15 (1.9)	1 (1.9)	
Hispanic	22 (2.8)	3 (5.7)	
White	545 (69.8)	38 (71.7)	
Other/Multiracial	100 (12.8)	7 (13.2)	
**Parental education (n=772; Not Classifiable: n=52)**			0.46
High school or less	118 (15.3)	11 (21.2)	
Trades/college	229 (29.7)	18 (34.6)	
Bachelor's degree	283 (36.7)	16 (30.8)	
Higher than Bachelor's degree	142 (18.4)	7 (13.5)	
**Social deprivation index (percentile), median (IQR) (n=760; Not Classifiable: n=158)**	40.0 (23.0, 65.0)	47.0 (22.2, 71.8)	0.13
**Material deprivation index (percentile), median (IQR) (n=760; Not Classifiable: n=158)**	26.0 (11.0, 54.0)	35.0 (12.2, 56.8)	0.11
**Past concussion maximum symptom duration (n=780; Not Classifiable: n=167)**			0.80
<1 week or no previous concussions	637 (81.7)	135 (80.8)	
1+ week(s)	143 (18.3)	32 (19.2)	
**History of migraines (n=777; Not Classifiable: n=168)**	42 (5.4)	15 (8.9)	0.08
**Family history of migraine headaches (n=785; Not Classifiable: n=55)**	291 (37.1)	24 (43.6)	0.33

© 2023 van Ierssel JJ et al. *JAMA Network Open.*

eTable 1. (cont'd)

Variable	Classifiable N=787 No. (%)	Not Classifiable N=180 No. (%)	p-value
**5P risk score at ED visit, median (IQR) (n=781; Not Classifiable: n=166)**	5.0 (4.0, 7.0)	5.0 (4.0, 6.0)	0.16
**Preinjury HBI score[b], median (IQR) (n=785; Not Classifiable: n=51)**			
Total	9.0 (3.0, 17.0)	13.0 (4.0, 22.0)	0.06
Cognitive	7.0 (2.0, 14.0)	8.0 (0.5, 15.0)	0.72
Somatic	1.0 (0.0, 4.0)	3.0 (1.0, 7.0)	0.001
**Preinjury PedsQL-4.0 score[b], median (IQR) (n=785; Not Classifiable: n=51)**			
Total	84.8 (75.0, 93.5)	82.6 (73.9, 89.1)	0.55
Physical activity	93.8 (84.4, 100.0)	93.8 (79.1, 100.0)	0.95
Emotional functioning	80.0 (65.0, 95.0)	75.0 (62.5, 92.5)	0.40
Social functioning	90.0 (75.0, 100.0)	90.0 (75.0, 100.0)	0.40
School functioning	80.0 (65.0, 90.0)	75.0 (65.0, 90.0)	0.87

Abbreviations: *ED*, emergency department; *HBI*, Health and Behaviour Inventory; *IQR*, interquartile range; *OI*, orthopaedic injury; *PedsQL-4.0*, Pediatric Quality of Life Inventory – Version 4.0.

[a] Participants were classified into headache phenotype based on symptoms collected at a post-acute visit targeted for 10 days post-injury (i.e., complete reporting of headache, nausea, photophobia, phonophobia).

[b] Parent-rated

© 2023 van Ierssel JJ et al. *JAMA Network Open.*

eTable 2. Total and Subscale Mean Differences on the Child-Reported HBI[a] at 3 Months Among Headache Phenotype Groups

Headache Phenotype	Estimated Mean Difference (95%CI)[b]	Adjusted p (Bonf-Holm)
**HBI Total score[c]**		
Concussion + PTH-M vs. Concussion + PTH-NM	1.93 (-0.33 to 4.19)	0.38
Concussion + PTH-M vs. Concussion + no-PTH	3.36 (1.13 to 5.60)	0.02
Concussion + PTH-M vs. OI	3.10 (0.75 to 5.44)	0.05
Concussion + PTH-NM vs. Concussion + no-PTH	1.44 (-0.95 to 3.82)	0.71
Concussion + PTH-NM vs. OI	1.17 (-1.29 to 3.63)	0.71
Concussion + no-PTH vs. OI	-0.265 (-2.60 to 2.07)	0.82
**HBI Cognitive subscore**		
Concussion + PTH-M vs. Concussion + PTH-NM	1.07 (-0.55 to 2.69)	0.78
Concussion + PTH-M vs. Concussion + no-PTH	1.96 (0.37 to 3.55)	0.10
Concussion + PTH-M vs. OI	1.99 (0.36 to 3.62)	0.10
Concussion + PTH-NM vs. Concussion + no-PTH	0.89 (-0.812 to 2.59)	0.89
Concussion + PTH-NM vs. OI	0.92 (-0.81 to 2.64)	0.89
Concussion + no-PTH vs. OI	0.03 (-1.61 to 1.67)	0.97
**HBI Somatic subscore**		
Concussion + PTH-M vs. Concussion + PTH-NM	0.839 (-0.11 to 1.79)	0.33
Concussion + PTH-M vs. Concussion + no-PTH	1.39 (0.43 to 2.36)	0.03
Concussion + PTH-M vs. OI	1.10 (0.06 to 2.14)	0.19
Concussion + PTH-NM vs. Concussion + no-PTH	0.55 (-0.48 to 1.56)	0.88
Concussion + PTH-NM vs. OI	0.26 (-0.81 to 1.33)	1.00
Concussion + no-PTH vs. OI	-2.9 (-1.34 to 0.75)	1.00

Abbreviations: *HBI*, Health and Behaviour Inventory; *ns*, non-significant; *OI*, orthopaedic injury; *PTH*, posttraumatic headache

[a] HBI Total range: 0-60; Cognitive range: 0-33; Somatic range: 0-27; Higher score indicates higher symptom burden.

[b] Estimated outcomes are adjusted to the median (continuous) or mode (categorical) of age, sex, race, parental education, social deprivation index, material deprivation index, past concussion maximum symptom duration, migraine history, family migraine history, 5P risk score, and preinjury HBI cognitive and somatic scores.

[c] Primary outcome

© 2023 van Ierssel JJ et al. *JAMA Network Open.*

eTable 3. Association of Posttraumatic Headache Phenotype With Quality of Life (PedsQL-4.0 Total score) at 3 Months in a Multivariable Linear Regression[a]

			PedsQL Total
**Parameter estimates**	**Coefficient (95% CI)**	***t***	**p-value**
**Intercept**	93.22 (87.69 to 98.74)	33.11	<.001
			**Headache phenotype**
Orthopaedic injury			Reference
Concussion + no-PTH	1.77 (-0.87 to 4.41)	1.32	0.19
Concussion + PTH-NM	-0.17 (-2.94 to 2.60)	-0.12	0.90
Concussion + PTH-M	-1.47 (-4.07 to 1.12)	-1.11	0.27
**Age**	0.05 (-0.30 to 0.40)	0.28	0.78
**Sex**			
Male			Reference
Female	-1.74 (-3.72 to 0.24)	-1.72	0.09
**Race**			
Asian	-2.39 (-5.39 to 0.61)	-1.57	0.12
Black	-2.93 (-7.47 to 1.60)	-1.27	0.20
Hispanic	-3.77 (-8.59 to 1.05)	-1.54	0.13
Indigenous	-0.44 (-4.21 to 3.32)	-0.23	0.82
White			Reference
Other/Multi-racial	-0.71 (-3.05 to 1.63)	-0.60	0.55
**Parental education**			
High school or less			Reference
Trades/college	-0.21 (-2.80 to 2.37)	-0.16	0.88
Bachelor’s degree	0.18 (-2.33 to 2.69)	0.14	0.89
Higher than Bachelor’s degree	1.23 (-1.65 to 4.11)	0.84	0.40
**Social deprivation index (percentile)**	-0.01 (-0.04 to 0.02)	-0.74	0.46
**Material deprivation index (percentile)**	-0.02 (-0.05 to 0.01)	-1.46	0.14
**Past concussion maximum symptom duration**			
<1 week/no previous concussions			Reference
1+ week(s)	1.50 (-0.68 to 3.68)	1.35	0.18
**Migraine history**	0.06 (-3.74 to 3.85)	0.03	0.98
**Family migraine history**	-1.23 (-2.97 to 0.51)	-1.39	0.16
**5P risk score at ED visit**	-0.82 (-1.40 to -0.23)	-2.74	0.006
**Preinjury HBI Cognitive subscore[b]**	-0.33 (-0.45 to -0.22)	-5.70	<.001
**Preinjury HBI Somatic subscore[b]**	-0.37 (-0.60 to -0.15)	-3.25	0.001

[a] Model L.R. χ^2=135.26 (df=21), p<0.001; R^2=0.14; Adj R^2=0.12; Observed=928
[b] Parent-reported

© 2023 van Ierssel JJ et al. *JAMA Network Open.*

eTable 4. Total and Subscale Mean Differences on the Child-Reported PedsQL-4.0[a] at 3 Months Among Headache Phenotype Groups

Headache Phenotype	Estimated Mean Difference (95%CI)[b]	Adjusted p (Bonf-Holm)
**PedsQL-4.0 Total score[c]**		
Concussion + PTH-M vs. Concussion + PTH-NM	-1.30 (-3.76 to 1.16)	0.80
Concussion + PTH-M vs. Concussion + no-PTH	-3.24 (-5.65 to -0.83)	0.05
Concussion + PTH-M vs. OI	-1.47 (-4.07 to 1.12)	0.80
Concussion + PTH-NM vs. Concussion + no-PTH	-1.94 (-4.6 to 0.71)	0.75
Concussion + PTH-NM vs. OI	-0.17 (-2.94 to 2.60)	0.90
Concussion + no-PTH vs. OI	1.77 (-0.87 to 4.41)	0.75
**PedsQL-4.0 Physical activity subscore**		
Concussion + PTH-M vs. Concussion + PTH-NM	-3.53 (-6.50 to -0.56)	0.10
Concussion + PTH-M vs. Concussion + no-PTH	-4.09 (-7.0 to -1.18)	0.04
Concussion + PTH-M vs. OI	-1.51 (-4.69 to 1.68)	0.71
Concussion + PTH-NM vs. Concussion + no-PTH	-0.56 (-3.78 to 2.66)	0.73
Concussion + PTH-NM vs. OI	2.02 (-1.28 to 5.33)	0.69
Concussion + no-PTH vs. OI	2.58 (-0.60 to 5.76)	0.44
**PedsQL-4.0 Emotional functioning subscore**		
Concussion + PTH-M vs. Concussion + PTH-NM	0.16 (-3.61 to 3.93)	1.00
Concussion + PTH-M vs. Concussion + no-PTH	-3.42 (-7.24 to 0.39)	0.47
Concussion + PTH-M vs. OI	-1.40 (-5.54 to 2.75)	1.00
Concussion + PTH-NM vs. Concussion + no-PTH	-3.59 (-7.78 to 0.61)	0.47
Concussion + PTH-NM vs. OI	-1.56 (-5.97 to 2.85)	1.00
Concussion + no-PTH vs. OI	2.03 (-2.10 to 6.15)	1.00
**PedsQL-4.0 Social functioning subscore**		
Concussion + PTH-M vs. Concussion + PTH-NM	2.26 (-0.86 to 5.38)	0.77
Concussion + PTH-M vs. Concussion + no-PTH	-1.51 (-4.50 to 1.47)	0.96
Concussion + PTH-M vs. OI	0.50 (-2.73 to -0.47)	0.96
Concussion + PTH-NM vs. Concussion + no-PTH	-3.78 (-7.08 to -0.47)	0.15
Concussion + PTH-NM vs. OI	-1.76 (-5.25 to 1.73)	0.96
Concussion + no-PTH vs. OI	2.02 (-1.27 to 5.30)	0.91
**PedsQL-4.0 School functioning subscore**		
Concussion + PTH-M vs. Concussion + PTH-NM	-2.73 (-6.20 to 0.74)	0.49
Concussion + PTH-M vs. Concussion + no-PTH	-3.42 (-6.90 to 0.07)	0.33
Concussion + PTH-M vs. OI	-3.46 (-7.13 to 0.21)	0.33
Concussion + PTH-NM vs. Concussion + no-PTH	-0.69 (-4.48 to 3.11)	1.00
Concussion + PTH-NM vs. OI	-0.73 (-4.67 to 3.20)	1.00
Concussion + no-PTH vs. OI	-0.04 (-3.77 to 3.68)	1.00

Abbreviations: *OI*, Orthopaedic injury; *PedsQL*, Pediatric Quality of Life Inventory - Version 4.0; *PTH*, Posttrauumatic headache
[a] PedsQL-4.0 transformed range for total and subscales: 0-100; Higher score indicates higher quality of life.
[b] Estimated outcomes are adjusted to the median (continuous) or mode (categorical) of age, sex, race, parental education, social deprivation index, material deprivation index, past concussion maximum symptom duration, migraine history, family migraine history, 5P risk score, and preinjury HBI cognitive and somatic scores.
[c] Minimal clinically meaningful difference for child-reported total and subscore change: total score: 4.36; physical score: 6.92; emotional score: 7.79; social score: 8.98; school change: 9.67 (reference: Varni JW, Burwinkle TM, Seid M, Skarr D. The PedsQL 4.0 as a pediatric population health measure: feasibility, reliability, and validity. Ambul Pediatr. 2003;3(6):329-341.)

© 2023 van Ierssel JJ et al. *JAMA Network Open.*
