# Supplementary material for: Association of Posttraumatic Headache With Symptom Burden After Concussion in Children
Source: JAMA Netw Open. 2023 Mar 8;6(3):e231993. doi: 10.1001/jamanetworkopen.2023.1993 (PMC9996395; doi:10.1001/jamanetworkopen.2023.1993)
Supplement: Supplement 2. — Pediatric Emergency Research Canada A-CAP Study Team Members [file jamanetwopen-e231993-s002.pdf]

Supplemental Online Content: Nonauthor Collaborators
*First name, last name, and suffix (if applicable) are required and will appear in PubMed.

*Group Name(s): Pediatric Emergency Research Canada A-CAP study team

*First Name and Middle Initial(s)	*Last Name	*Suffix (eg, Jr, III)	Academic Degrees	Institution	Location (city, state/province, country)	Role or Contribution, eg, chair, principal investigator	Group (if more than 1 Group listed in the byline) and/or Subgroup (eg, Steering Committee)
Bruce H	Bjornson		MD, BSc	Department of Pediatrics, University	Vancouver, British Columbi	Co-site PI	
Angelo	Mikrogianakis		MD	Department of Pediatrics, McMaster	Hamilton, Ontario, Canada	Co-investigator	
Bradley	Goodyear		PhD	Hotchkiss Brain Institute and Departr	Calgary, Alberta, Canada	Co-investigator	
Nishard	Abdeen		MD	Department of Radiology, University	Ottawa, Ontario, Canada	Collaborator	
Christian	Beaulieu		PhD	Department of Biomedical Engineerir	Edmonton, Alberta, Canada	Co-investigator	
Mathieu	Dehaes		PhD	Department of Radiology, Radioonco	Montreal, Québec, Canada	Collaborator	
Sylvain	Deschenes		PhD	Department of Radiology, Radioonco	Montreal, Québec, Canada	Collaborator	
Ashley	Harris		PhD	Hotchkiss Brain Institute and Alberta	Calgary, Alberta, Canada	Co-investigator	
Catherine	Lebel		PhD	Alberta Children's Hospital Research	Calgary, Alberta, Canada	Co-investigator	
Ryan	Lamont		PhD	Alberta Children's Hospital Research	Calgary, Alberta, Canada	Co-investigator	
Tyler	Williamson		PhD	Department of Community Health Sc	Calgary, Alberta, Canada	Collaborator	
Brian L	Brooks		PhD	Alberta Children's Hospital Research	Calgary, Alberta, Canada	Co-investigator	
Carolyn	Emery		PT, PhD	Hotchkiss Brain Institute and Alberta	Calgary, Alberta, Canada	Co-investigator	
Stephen B	Freedman		MD, MSc	Alberta Children's Hospital Research	Calgary, Alberta, Canada	Co-investigator	
Lianne	Tomfohr-Madsen		PhD	Faculty of Education, Univeristy of Br	Vancouver, British Columbi	Co-investigator	
Kathryn J	Schneider		PT, PhD	Hotchkiss Brain Institute and Alberta	Calgary, Alberta, Canada	Co-investigator	
