# Supplementary material for: Association of Posttraumatic Headache With Symptom Burden After Concussion in Children
Source: JAMA Netw Open. 2023 Mar 8;6(3):e231993. doi: 10.1001/jamanetworkopen.2023.1993 (PMC9996395; doi:10.1001/jamanetworkopen.2023.1993)
Supplement: Supplement 3. — Data Sharing Statement [file jamanetwopen-e231993-s003.pdf]

Data Sharing Statement

van Ierssel. Association of Posttraumatic Headache With Symptom Burden After Concussion in Children. *JAMA Netw Open*. Published March 08, 2023.
doi:10.1001/jamanetworkopen.2023.1993

Data
Data available: No

Additional Information
Explanation for why data not available: A dataset with deidentified participant data and a data dictionary will be made available upon reasonable request from any qualified investigator, subject to a signed data access agreement.
